# Impact of Co-occurring Mental Disorders and Chronic Physical Illnesses on Frequency of Emergency Department Use and Hospitalization for Mental Health Reasons

**DOI:** 10.3389/fpsyt.2021.735005

**Published:** 2021-11-22

**Authors:** Lia Gentil, Guy Grenier, Xiangfei Meng, Marie-Josée Fleury

**Affiliations:** ^1^Douglas Mental Health University Institute Research Centre, Montréal, QC, Canada; ^2^Department of Psychiatry, McGill University, Montréal, QC, Canada

**Keywords:** co-occurring mental disorders, chronic physical illnesses, emergency department (ED) use, hospitalization, clinical variables, sociodemographic variables, service use variables

## Abstract

**Background:** Patients with mental disorders (MD) are at high risk for a wide range of chronic physical illnesses (CPI), often resulting in greater use of acute care services. This study estimated risk of emergency department (ED) use and hospitalization for mental health (MH) reasons among 678 patients with MD and CPI compared to 1,999 patients with MD only.

**Methods:** Patients visiting one of six Quebec (Canada) ED for MH reasons and at onset of a MD in 2014–15 (index year) were included. Negative binomial models comparing the two groups estimated risk of ED use and hospitalization at 12-month follow-up to index ED visit, controlling for clinical, sociodemographic, and service use variables.

**Results:** Patients with MD, more severe overall clinical conditions and those who received more intensive specialized MH care had higher risks of frequent ED use and hospitalization. Continuity of medical care protected against both ED use and hospitalization, while general practitioner (GP) consultations protected against hospitalization only. Patients aged 65+ had lower risk of ED use, whereas risk of hospitalization was higher for the 45–64- vs. 12–24-year age groups, and for men vs. women.

**Conclusion:** Strategies including assertive community treatment, intensive case management, integrated co-occurring treatment, home treatment, and shared care may improve adequacy of care for patients with MD-CPI, as well as those with MD only whose clinical profiles were severe. Prevention and outreach strategies may also be promoted, especially among men and older age groups.

## Introduction

Emergency department (ED) and inpatient hospital services are among the costliest forms of healthcare (1, 2), often serving as a barometer for the quality of healthcare systems (3–5). Frequent ED use or hospitalization may reflect poor access, continuity, or inappropriate outpatient care (3, 6). Patients with mental disorders (MD) including substance-related disorders (SRD) and with chronic physical illnesses (CPI) are high ED users, which contributes substantially to ED overcrowding (7–9). They are also hospitalized and readmitted more often than patients without MD or CPI (10–13). Co-occurring CPI frequently occurs among patients with MD (14–18). Mental disorders-chronic physical illnesses are associated with patient disability (19), social dysfunction (20), treatment complications (21), poor quality of life (19), and higher risk of mortality (22). Patients with MD-CPI also face challenges in seeking care (6, 23), as healthcare systems tend to favor treatment of individual diseases rather than multimorbidity, whereas treating MD and CPI in isolation is generally ineffective (24, 25). As well, CPI in patients with MD are often under-diagnosed or undertreated (18, 23), particularly among patients with serious MD-CPI making limited use of primary care (23, 26, 27). Other care-related challenges involve socio-economic barriers (28) and social stigma (29). General practitioners (GP) are usually viewed as inadequately equipped to treat more serious or complex MD (28, 30).

The overall, combined effect of acute care service use for mental health (MH) reasons among patients with MD and CPI, compared with MD only, has rarely been assessed. Studies have more often investigated ED use and hospitalization for medical reasons. Of the few studies that compared MD-CPI with MD or CPI only, most found that patients with MD-CPI were more likely than those with CPI or MD only to use ED frequently (31–33), to require hospitalization (34) and to face longer duration of hospital stay (32). Moreover, numerous studies have compared ED use and hospitalization for MH reasons, but only in terms of specific CPI and MD (31, 33, 35), e.g., diabetes and schizophrenia or serious MD (31, 33, 35), cancer and common MD (32), epilepsy and MD (36), migraine and MD (34), and CPI and MD among self-harming adolescents (25).

Most studies comparing patients with MD-CPI to those with MD or CPI only have controlled for few clinical and socio-demographic variables (18, 25, 33–37). Results showed that the risk of frequent ED use and hospitalization increased with severity of CPI in patients with serious MD (24, 33) or depressive disorders (18). Being a woman, younger and living in poorer neighborhoods reportedly increased ED use among patients with co-occurring schizophrenia and diabetes (31). Overall, this literature tended to omit assessments of service use variables, although some studies did find that patients with MD-CPI and more frequent ED users were also more likely to use outpatient services, take medications (34, 38) and to be covered by health insurance (36).

Better knowledge of how MD-CPI, vs. MD only, impact ED use and hospitalization for MH reasons, controlling for service use, may contribute to improvement in health service management (39). Adequate intensity, continuity, and diversity of care for patients with CPI or MD and access to a family doctor or psychiatrist, particularly among patients with MD-CPI, could lower rates of ED use or preventable hospitalization. This study aimed to compare frequencies of ED use and hospitalization for MH reasons among patients with MD only compared to patients with MD-CPI, controlling for clinical, sociodemographic, and service use variables. We hypothesized that patients with MD-CPI would make more frequent use of ED and experience more frequent hospitalization for MH reasons, and that both (1) severity of MD-CPI and (2) less intensity, diversity, or continuity of service use would increase frequency of ED use and hospitalization.

## Methods

### Study Context

This study was conducted in the province of Quebec, which accounts for 22% of the Canadian population. Responsibility for Quebec health and social services is integrated within a single ministry. MH is one of nine service programs under the *Quebec Ministère de la Santé et des Services sociaux* (Ministry of Health and Social Services) (40). A major health reform in 2015 led to the creation of 22 integrated health and social services centers resulting from the merger of nearly all health and social institutions, like hospitals, community healthcare centers, and nursing homes, within each of the networks. Specialized MH services in the integrated networks are offered in psychiatric departments of general hospitals or in psychiatric hospitals. Public primary care MH services are offered by GP working in private medical clinics, in most cases family medicine groups. Over 60% of Quebec GP work in these group practices, benefitting from added psychosocial clinicians like nurses and social workers and enhanced secretarial support. Family medicine groups insure patient registration, better access to care, and care continuity through expanded days and hours of medical coverage, including walk-in clinics (41). Primary MH care is also provided in community healthcare centers offering mainly psychosocial interventions, although some GP also work there on a salaried basis, whereas most physicians are remunerated on a fee-for-service basis. Community MH organizations (e.g., crisis centers, self-help groups) and psychologists working in private practice complete the Quebec MH system.

### Study Population and Design

This 4-year cohort study (2012–13 to 2015–16) investigated 12,000 patients identified through provincial medical administrative databases who visited at least one of six Quebec ED in 2014–15 (index year) for MH reasons, including SRD. Only patients at onset of a MD at index ED visit, i.e., incident cases not diagnosed with MD in the previous 2 years, were included in the study (*n* = 2,819). Of these patients, 142 who were diagnosed with incident CPI after the date of their index ED visit in 2014–2015 (90 participants) were excluded from the study, as were those hospitalized for more than 90 days following the index ED visit (42), because outpatient care for these patients over the 12-month follow-up period would not be adequately assessed. The final sample thus consisted of 2,677 patients, 12+ years old and eligible for health insurance under the Quebec health care regime [*Régie de l'Assurance Maladie du Québec* (RAMQ)]. Patients were further divided into two groups: those with MD-CPI (*n* = 678: 25%) or MD only (*n* = 1,999: 75%). Chronic physical illnesses were identified for a 2-year period preceding index ED visits in 2014–15, at which time incident cases were diagnosed (43). Six ED located in major Quebec cities within University or peripheral health regions were selected for the study. The Quebec Access to Information Commission authorized the study and the ethics committee of a MH University institute approved the study protocol.

### Data Sources

Data for the study were obtained from the RAMQ database, which contains Quebec medical administrative data including billing files for medical services provided by physicians on a fee-for-service basis, patient diagnoses, and patient sociodemographic information including material and social deprivation indices. Only 6% of billing occurred outside the public system in 2016–17 (RAMQ, 2020). Hospitalization data were obtained from the “*Maintenance et exploitation de données pour l'étude de la clientèle hospitalière*” (Med-Echo) database. Emergency department data (e.g., reasons for ED visits) were provided by the “*Banque de données commune des urgences*” (BDCU) database, while the “*Système d'information clinique et administrative des centres locaux de services communautaires*” (I-CLSC) database provided complementary data on psychosocial interventions and medical care provided by salaried GP in community healthcare centers. Data from these sources were merged for each patient using a unique encrypted identifier.

### Variables

The dependent variables were frequency of ED use and frequency of hospitalization for MH reasons at 12 months following index ED visits in 2014–2015, with MH including SRD and suicidal behaviors (e.g., ideation, attempt). Emergency department use or hospitalization for physical conditions (primary or secondary diagnoses) were excluded. Main independent variables were the two groups compared: MD-CPI and MD only. Control variables included clinical, sociodemographic, and service use variables. Clinical variables involved MD, SRD, and CPI, with severity levels (0–3+) based on the Elixhauser Comorbidity Index (44, 45). Mental Disorders included common MD (adjustment, depressive, and anxiety disorders), serious MD (schizophrenia spectrum and other psychotic disorders, bipolar disorders), and personality disorders, while SRD included alcohol and drug use and induced disorders, intoxication, and withdrawal. Chronic physical illnesses consisted of major illness categories (e.g., hypertension, liver or valvular illnesses, coagulopathy). The four diagnoses categories involving MD and SRD were excluded from the original Elixhauser Comorbidity Index list of 31 CPI (44, 46). Diagnostic codes for MD, SRD, and CPI in the RAMQ were based on the International Classification of Diseases Ninth Revision (ICD-9) and those in the MED-ECHO and BDCU on the Tenth Revision (ICD-10-CA) (Table 1). These RAMQ codes for case definition have good reliability and sensitivity (43, 47, 48).

**Table 1:** Codes for mental disorders (MD), substance-related disorders (SRD), and chronic physical illnesses (CPI) according to the International Classification of Diseases, Ninth and Tenth revisions^a^.

**Diagnoses**	***International Classification of Diseases, Ninth Revision (ICD-9)***	***International Classification of Diseases, Tenth Revision, Canada* (*ICD-10-CA)***
***Mental disorders (MD)***		
***Common MD***		
Adjustment disorders	309.0–309.4, 309.8, 309.9	F43.0–F43.2; F43.8, F43.9
Depressive disorders	300.4, 311.9	F32.0–F32.8, F32.9, F33.0; F33.8, F33.9, F34.8, F34.9, F38.0, F38.1, F38.8, F39, F41.2
Anxiety disorders	300 (except 300.4)	F40–F48, F68
***Serious MD***		
Schizophrenia spectrum and other psychotic disorders	295, 297, 298	F20, F21, F22, F23, F24, F25, F28, F29, F32.3; F33.3; F44.89
Bipolar disorders	296.0–296.6, 296.8, 296.9	F30.0–F30.2, F30.8, F30.9, F31.0–F31.9
***Personality disorders***	301	F60, F07.0, F34.0, F341, F48.8, F61
***Substance-related disorders (SRD)***		
Alcohol-related disorders	291.0, 291.8 (alcohol withdrawal); 303.0, 303.9, 305.0 (alcohol abuse or dependence); 291.1–291.5, 291.9, 357.5, 425.5, 535.3, 571.0–571.3 (other alcohol-induced disorders); 980.0, 980.1, 980,8, 980.9 (alcohol intoxication)	F10.1, F10.2 (alcohol abuse or dependence); F10.3, F10.4 (alcohol withdrawal); F10.5–F10.9, K70.0–K70.4, K70.9, G62.1, I42.6, K29.2, K85.2, K86.0, E24.4, G31.2, G72.1, O35.4 (other alcohol-induced disorders); F10.0, T51.0, T51.1, T51.8, T51.9 (alcohol intoxication)
Drug-related disorders	292.0 (drug withdrawal); 304.0–304.9, 305.2–305.7, 305.9 (drug abuse or dependence); 292.1, 292.2, 292.8, 292.9 (other drug-induced disorders)965.0, 965.8, 967.0, 967.6, 967.8, 967.9, 969.4–969.9, 970.8, 982.0, 982.8 (drug intoxication)	F11.1, F12.1, F13.1, F14.1, F15.1, F16.1, F18.1, F19.1, F11.2, F12.2, F13.2, F14.2, F15.2, F16.2, F18.2, F19.2, F55 (drug abuse or dependence);); F11.3–F11.4, F12.3, F12.4; F13.3–F13.4, F14.3–F14.4, F15.3–F15.4, F16.3–F16.4, F18.3–F18.4, F19.4–F19.4 (drug withdrawal); F11.5–F11.9, F12.5–F12.9; F13.5–F13.9, F14.5–F14.9, F15.5–F15.9, F16.5–F16.9, F18.5–F18.9, F19.5–F19.9 (other drug-induced disorders; F11.0, F12.0, F13.0, F14.0, F15.0, F16.0, F18.0, F19.0, T40.0–T40.9, T42.3, T42.4, T42.6, T42.7, T43.5, T43.6, T43.8, T43.9, T50.9, T52.8, T52.9 (drug intoxication)
***Chronic physical illnesses (CPI)***		
Renal failure	403.0, 403.1, 403.9, 404.0, 404.1, 404.9, 585, 586, 588.0, V42.0, V45.1, V56	I12.0, I13.1, N18.x, N19.x, N25.0, Z49.x, Z94.0, Z99.2
Cerebrovascular illnesses	362.34, 430.x−438.x	G45.x, G46.x, I60.x–I69.x
Neurological illnesses	331.9, 332.0, 332.1, 333.4, 333.5, 333.9, 334–335, 336.2, 340, 341, 345, 348.1, 348.3, 780.3, 784.3	G10.x–G12.x, G13.x, G20.x, G21.x–G22.x, G25.4, G25.5, G31.8, G31.9, G32.x, G35.x, G36.x, G37.x, G40.x, G41.x, G93.1, G93.4, R47.0, R56.x
Endocrine illnesses (excluding diabetes mellitus)	240.9, 243.x, 244.x, 246.1, 246.8, 278.0, 253.6, 276.x	E66.x, E00.x, E01.x, E02.x, E03.x, E89.0, E22.2, E86.x, E87.x
Any tumor and metastatic cancer	140.x−172.x, 174.x, 175.x, 179.x−195.x, 196.x−199.x, 200.x, 201.x, 202.x, 203.0, 238.6, 273.3	C00.x–C26.x, C30.x–C34.x, C37.x–C41.x, C43.x, C45.x–C58.x, C60.x–C76.x, C77.x–C79.x, C80.x, C81.x–C85.x, C88.x, C90.0, C90.2, C96.x
Chronic pulmonary illnesses	490x−505.x, 506.4, 508.1, 508.8	I27.8, I27.9, J40.x–J47.x, J60.x–J64.x, J65.x, J66.x, J67.x, J68.4, J70.1, J70.3
Diabetes complicated and uncomplicated	250.0–250.2, 250.3, 250.4–250.9	E10.2–E10.8, E11.2–E11.8, E13.2–E13.8, E14.2–E14.8, E10.0, E10.1, E10.9, E11.0, E11.1, E11.9, E13.0, E13.1, E13.9, E14.0, E14.1, E14.9
Cardiovascular illnesses	394–397, 424, 746.3–746.6, V42.2, V43.3, 401, 402–405, 437.2, 398.9, 402.0, 402.1, 402.9, 410, 412, 415.0, 415.1, 416, 417.0, 417.8, 417.9, 428, 426.0, 426.1, 426.5–426.7, 426.9, 427.0–427.4, 427.6–427.9, 437.2, 785.0, 996.0, V45.0, V53.3, 093, 440, 441, 443.1–443.9, 447.1, 557.1, 557.9, V43.4	I05.x–I08.x, I09.1, I09.8, I10.x, I11.x–I13.x, I15.x, I67.4, I09.9, I11.0, I13.0, I13.2, I21.x, I22.x, I25.2, I25.5, I26.x, I27.x, I28.0, I28.8, I28.9, I34.x–I39.x, I42.0, 142.5I42.9, I43.x, I50.x, P29.0, I44.1–I44.3, I45.6, I45.9, I47.x–I49.x, Q23.0–Q23.3, Q23.8, Q23.9 R00.0, R00.1„ R00.8, T82.1, Z45.0, Z95.0, Z95.2, Z95.3, Z95.4, A52.0, I70.x, I71.x,I72.x, I73.0, I73.1, I73.8, I73.9, I77.1, I79.0, K55.1, K55.8, K55.9, Z95.8, Z95.9
Other CPI categories (blood loss anemia, ulcer, liver illnesses, AIDS/HIV, rheumatoid arthritis/collagen vascular illnesses, coagulopathy, weight loss, paralysis, deficiency anemia)	280.0, 280.90, 286.x, 287.1, 287.3–287.5 531.7, 531.9, 532.7, 532.9, 533.7, 533.9, 534.7, 534.9, 070.22, 070.23, 070.2, 070.32, 070.33, 070.3, 070.44, 070.4, 070.54, 070.5, 456.0–456.2, 572.3, 572.8, 573.3, 573.4, 573.9, V42.7, 042.x−044.x, 136.1, 446.x, 701.0, 710.0–710.4, 710.5, 710.8, 710.9, 711.2, 714.x, 719.3, 720.x, 725.x, 728.5, 728.8, 729.30, 260.x−263.x, 783.2, 799.4, 334.1, 342.x, 343.x, 344.0, 344.1, 344.2, 344.3, 344.4, 344.5, 344.6, 344.8, 344.9, 280.1–289.0, 280.91–280.0 281.x, 285.9,	B20.x–B24.x, D50.0, D65–D68.x, D69.1, D69.3–D69.6 K25.7, K25.9, K26.7, K26.9, K27.7, K27.9, K28.7, K28.9, B18.x, I85.x, I86.4, I98.2, K71.1, K71.3–K71.5, K71.6, K71.7, K72.1, K72.9, K73.x, K74.x, K75.4, K76.0, K76.1, K76.3, K76.4, K76.5, K76.6, K76.8, K76.9, Z94.4, L90.0, L94.0, L94.1,L94.3, M05.x, M06.x, M08.x, M12.0, M12.3, M30.x, M31.x, M32.x–M35.x, M45.x, M46.0, M46.1, M46.8, M46.9x, G04.1, G11.4, G80.x, G81.x, G82.x, G83.x, E40.x–E46.x, R63.4, R64.x, D50.1, D50.8, D50.9, D51.x–D53.x, D63.x, D64.9

a *MD, SRD, and CPI identified in RAMQ (Régie de L'assurance Maladie du Québec) were based on the International Classification of Diseases Ninth Revision (ICD-9), while MED-ECHO (hospitalization database: Maintenance et exploitation de données pour l'étude de la clientèle hospitalière) and BDCU (emergency department database: Banque de Données Commune des Urgences) were based on the ICD Tenth Canadian Revision (ICD-10-CA)*.

Sociodemographic variables measured at index ED visits included age, sex, and material and social deprivation. Deprivation indices were calculated based on individual place of residence as determined by postal code and reported in the 2011 Canadian census. The Material Deprivation Index measures the ratio of population employment, average income, and number of individuals without a high school diploma for a given area; while the Social Deprivation Index calculates proportions of individuals living alone, without spouse, and single-parent families (49). Both indices are classified in quintiles, the fifth representing highest level of deprivation. In this study, material and social deprivation were regrouped and tested into three groups: least deprived (quintiles 1–2), moderately deprived (quintile 3), and most deprived (quintiles 4–5) or with no assignment, including individuals who were homeless, incarcerated, or living in institutions such as nursing homes.

Service use variables measured at 12-month follow-up were controlled for intensity, continuity, and diversity of patient care, as possible influences on ED use and hospitalization. These variables included frequency of consultations with usual GP, or usual outpatient psychiatrist; main physician provider (none, GP only, psychiatrist only, both GP, and psychiatrist); continuity of physician care; and number of psychosocial interventions in community healthcare centers. To qualify as “usual GP,” at least two consultations had to have been made with a single GP, or at least two consultations with more than one GP working in the same family medicine group (50). Regarding “usual psychiatrist,” patients who made only one outpatient psychiatric consultation had to have made at least two consultations with their GP, referred to as collaborative care (51). Continuity of physician care was measured with the Usual Provider Continuity Index, which described the proportion of visits to the usual GP and usual outpatient psychiatrist of total visits made (52), with a score ≥0.61 as the cut-off for high continuity of care (53).

### Data Analyses

Descriptive analyses were performed comparing the two main independent variables (patients with MD-CPI and MD only) and sociodemographic, clinical, and service use control variables and both dependent variables (or outcomes: ED use and hospitalization). Frequencies and percentages were calculated for all variables. As missing values were fewer than 0.5%, complete case analysis was used (54). The Intraclass Correlation Coefficient (ICC) was small (0.036), indicating low correlation among patients in hospital settings making further multilevel analysis unnecessary. Intercorrelation tests were also conducted, analyzing associations between each independent and control variable; those with correlation coefficients >0.7 were eliminated. Negative binomial models were developed to explore differences between the two main independent variables (MD-CPI and MD only) on the outcomes, frequency of ED use, and frequency of hospitalization for MH reasons at 12-month follow-up to the 2014–2015 index visit, controlling for key clinical, sociodemographic, and service use variables. Negative binomial analysis showed better goodness-of-fit than results for the zero-inflated models. Based on the Akaike's Information Criterion (AIC) and Bayesian Information Criterion (BIC), models with the smallest AIC or BIC were chosen as the final multivariate models. Interactions between sex and patient group were not significant. Data analysis was performed using STATA 17.0 software (54).

## Results

**Figure 1:**
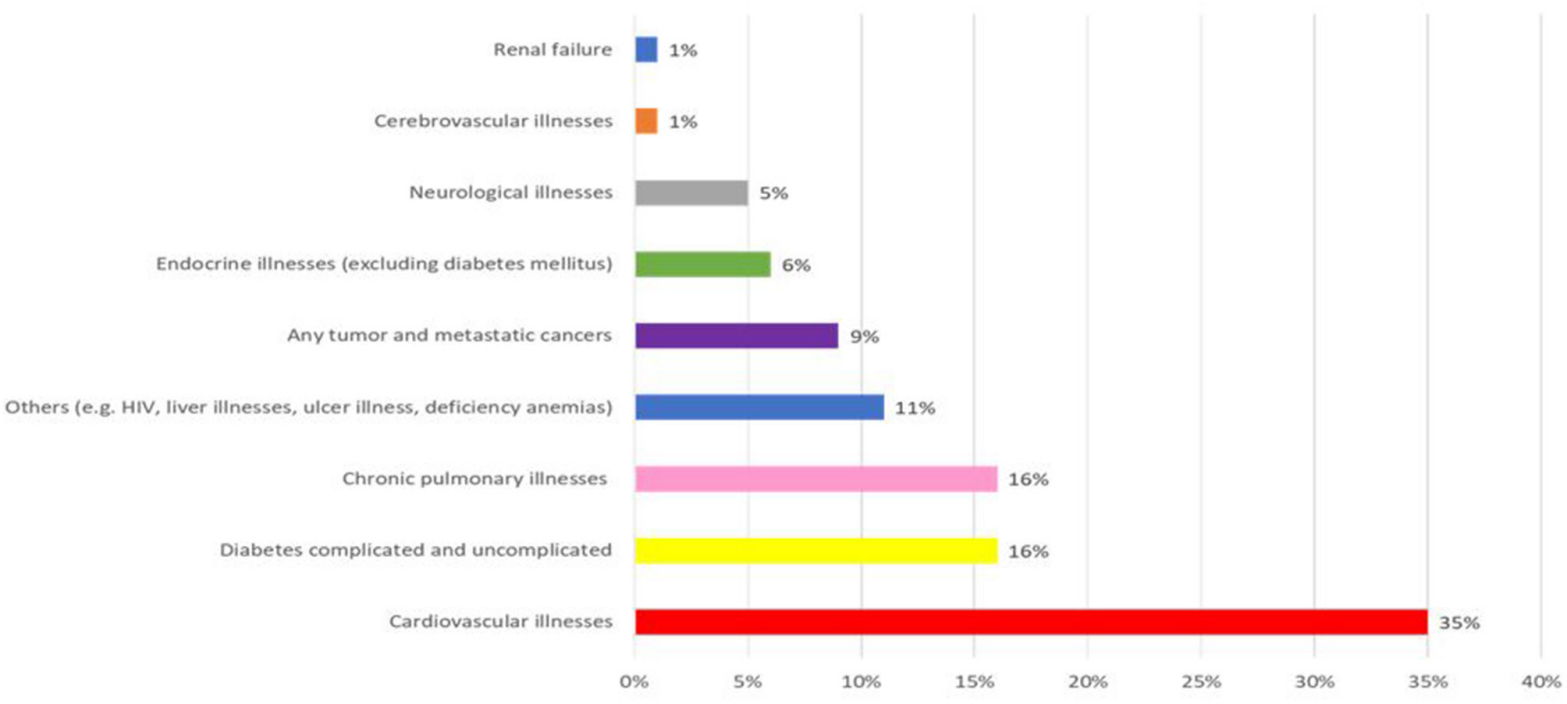
Prevalence of chronic physical illnesses (CPI) major health categories. **Cardiovascular illnesses** included: hypertension, myocardial infraction, congestive heart failure, cardiac arrhythmias, valvular illnesses, peripheral vascular illnesses; **diabetes** included: diabetes mellitus type 2 and diabetes mellitus type 1 with systemic manifestations and without mention of complications; **chronic pulmonary illnesses** included: chronic obstructive pulmonary illnesses, pnemoconiosis, and lung illnesses due to external agents; **other CPI** included: blood loss anemia, ulcer, liver illnesses, AIDS/HIV, rheumatoid arthritis/collagen vascular illnesses, coagulopathy, weight loss, paralysis, deficiency anemia; **any tumor and metastatic cancers** including malignant neoplasm with/without metastasis; **endocrine illnesses** included: hypothyroidism, fluid, and electrolyte illnesses, obesity; **neurological illnesses** included: paralysis agitans, secondary Parkinsonism, Huntington's chorea, other chorea, spinocerebellar illnesses, anterior horn cell illnesses, multiple sclerosis, other demmyelinating diseases of the central nervous system, convulsions, aphasia; **cerebrovascular illnesses** included: ocular ischemic syndrome, subarachnoid hemorrhage, intracerebral hemorrhage, other or unspecified intracranial hemorrage, transient cerebral ischemia, acute but ill-defined cerebrovascular illnesses, late effects of cerebrovascular illnesses; **renal failure** included: hypertensive renal disease, chronic renal failure, unspecified renal failure.

At 12-month follow-up, 10% of the cohort (95% CI: 9–11) had made no ED visits, 51% (95% CI: 48–52) only one, and 39% (95% CI: 37–40) two or more visits for MH reasons, with a mean of 1.82 visits (SD: 2.08; range: 0–26; median: 1; IQR: 1), while 77% were not hospitalized (mean: 0.29; SD: 0.62; range: 0–9; median: 0; IQR:1). Of the 2,677 patients, 52% (95% CI: 50–54) had common MD, 19% (95% CI: 17–20) serious MD, 12% (95% CI: 11–13) personality disorders, and 21% (95% CI: 20–23) SRD (Table 2). Regarding CPI, cardiovascular illnesses (35%; 95% CI: 33–37) were most prevalent, followed by chronic pulmonary illnesses, and complicated or uncomplicated diabetes (16% each; 95% IC: 14–18) (Figure 1). However, CPI severity index were low, with 89% (95% CI: 87–90) having a score of 0. For this cohort, 37% (95% CI: 35–39) of patients were 25–44 years old and 52% (95% CI: 50–54) were men. Ratings of 4 and 5 or not assigned, in the Material and Social Deprivation Indices were 45% (95% CI: 43–47) for material deprivation and 62% (95% CI: 60–64) for social deprivation. Following index ED visits, 51% (95% CI: 49–52) and 68% (95% CI: 67–70) reported no consultation with their usual GP or psychiatrist; 37% (CI: 35–39) of the sample had no main physician provider (either GP or psychiatrist), while GP were assigned as the main providers for 31% (95% CI: 29–33%). For the entire sample, 41% (95% CI: 39–42) received high scores for continuity of physician care, and 30% (95% CI: 28–33) for psychosocial interventions in community healthcare centers.

**Table 2:** Characteristics of patients at onset of a mental disorder (MD) including substance-related disorders (SRD) who used emergency departments (ED) in the index year 2014–15.

**Characteristics**	**Study sample patients**		**MD and chronic physical** **illnesses (CPI) group**		**MD group only**	
	***N***	**% (95% CI)**	***N***	**% (95% CI)**	***N***	**% (95% CI)**
Overall	2,677	100	678		1,999	100
**Clinical variables** (2012–13 to index ED visit)						
Mental disorders (MD)^a^						
Common MD	1,399	52.3 (50.3–54.1)	335	49.4 (45.6–53.1)	1,064	53.2 (51.0–55.4)
Serious MD	499	18.6 (17.2–20.1)	148	21.8 (18.8–25.1)	351	17.6 (15.9–19.2)
Personality disorders	324	12.1 (10.9–13.3)	67	9.9 (7.8–12.3)	257	12.9 (11.4–14.3)
Substance-related disorders (SRD)	568	21.2 (19.7–22.8)	144	21.2 (18.3–24.4)	424	21.2 (19.4–23.0)
Elixhauser Comorbidity Index (severity of chronic physical illnesses—CPI)^b^						
0	2,374	88.6 (87.4–89.8)	375	55.3 (51.5–59.0)	1,999	100
1	119	4.4 (3.7–5.2)	119	17.6 (14.8–20.6)	0	0
2	75	2.8 (2.2–3.4)	75	11.1 (8.9–13.6)	0	0
3+	109	4.0 (3.3–4.8)	109	16.1 (13.4–19.0)	0	0
**Sociodemographic variables** (2014–15)						
Age						
12–24 years	767	28.7 (26.9–30.3)	69	10.2 (8.1–12.6)	698	34.9 (32.8–37.0)
25–44 years	995	37.2 (35.3–39.0)	173	25.5 (22.3–28.9)	822	41.1 (38.9–43.2)
45–64 years	702	26.1 (24.5–27.9)	273	40.3 (36.6–44.0)	429	21.5 (19.7–23.3)
65+ years	213	7.9 (6.9–9.0)	163	24.0 (20.9–27.4)	50	2.5 (1.9–3.2)
Sex						
Men	1,395	52.1 (46.0–49.7)	330	48.7 (44.9–52.4)	1,065	53.3 (51.0–55.4)
Women	1,282	47.8 (50.2–54.0)	348	51.3 (47.5–55.0)	934	46.7 (44.5–48.9)
Material Deprivation Index						
1–2: least deprived	984	36.8 (34.9–38.6)	229	33.8 (30.3–37.4)	755	37.8 (35.6–39.9)
3	481	18.0 (16.5–19.4)	121	17.8 (15.1–20.9)	360	18.0 (16.3–19.7)
4–5 most deprived or not assigned^c^	1,212	45.2 (43.3–47.1)	328	48.4 (44.6–52.1)	884	44.2 (42.0–46.4)
Social Deprivation Index						
1–2: least deprived	633	23.3 (22.0–25.2)	151	22.2 (19.2–25.5)	482	24.1 (22.2–26.0)
3	387	14.5 (13.1–15.8)	92	13.6 (11.1–16.3)	295	14.8 (13.2–16.3)
4–5: most deprived or not assigned^c^	1,657	61.8 (60.0–63.7)	435	64.2 (60.4–67.6)	1,222	61.1 (58.9–63.2)
**Service use variables** (12-month follow-up to index ED visit in 2014–15)						
Frequency of consultations with usual general practitioner (GP)^d^						
0 consultation	1,354	50.6 (48.6–52.4)	257	37.9 (34.3–41.6)	1,097	54.9 (52.6–57.0)
1–3 consultations	622	23.2 (21.6–24.8)	181	26.7 (23.4–30.1)	441	22.1 (20.2–23.9)
4+ consultations	701	26.2 (24.5–27.8)	240	35.4 (31.8–39.0)	461	23.0 (21.2–24.9)
Frequency of consultations with usual outpatient psychiatrist^e^						
0 consultation	1,830	68.4 (66.5–70.0)	452	66.7 (63.0–70.1)	1,378	68.9 (66.8–70.9)
1–3 consultations	385	14.4 (13.1–15.7)	91	13.4 (11.0–16.2)	294	14.7 (13.2–16.3)
4+ consultations	462	17.3 (15.8–18.7)	135	19.9 (17.0–23.0)	327	16.4 (14.7–18.0)
Main physician provider^f^						
GP only	834	31.1 (29.4–32.9)	275	40.6 (36.9–44.3)	559	28.0 (26.0–29.9)
Psychiatrist only	347	13.0 (11.7–14.2)	66	9.7 (7.7–12.2)	281	14.0 (12.6–15.6)
Both GP and psychiatrist	500	18.7 (17.2–20.1)	160	23.6 (20.5–26.9)	340	17.0 (15.4–15.6)
None (neither GP nor psychiatrist)	996	37.2 (35.3–39.0)	177	26.1 (22.9–29.5)	819	41.0 (38.8–43.1)
Usual Provider Continuity Index integrating both GP and psychiatrist (continuity of physician care)^g^						
≥0.61 (high continuity of physician care)	1,085	40.5 (38.6–42.4)	226	33.3 (29.8–36.9)	859	43.0 (40.8–45.1)
Frequency of psychosocial interventions in community healthcare centers (excluding GP interventions)						
0 intervention	1,869	69.8 (68.0–71.5)	507	74.7 (71.3–77.9)	1,362	68.1 (66.0–70.1)
1–3 interventions	463	17.3 (15.9–18.7)	92	13.6 (11.1–16.3)	371	18.6 (16.9–20.3)
4+ interventions	345	12.9 (11.6–14.2)	79	11.6 (9.4–14.2)	266	13.3 (11.8–14.8)

*CI, confidence interval*.

a *Patients may have made more than one ED visit for MD in index year 2014–15, so total percentage may exceed 100%*.

b *CPI were diagnosed 2 years prior to index ED visit in 2014–15. Elixhauser Comorbidity Index included: chronic pulmonary illnesses, cardiac arrhythmias, tumor w/o metastasis, renal failure, fluid electrolyte illnesses, myocardial infarction, congestive heart failure, metastatic cancer, dementia, cerebrovascular illnesses, neurological illnesses, liver illnesses (excluding alcohol-induced liver disease), pulmonary circulation illnesses, coagulopathy, weight loss, paralysis, AIDS/HIV. Of the 31 illnesses reported in this index, the 4 related to MD or SRD categories were excluded. This index was originally developed for mortality, and not for ED use or hospitalization, which may have impacted findings for this study*.

c *Missing address or living in an area where index assignment is not available. An index cannot usually be assigned to residents of nursing homes or homeless individuals*.

d *Usual GP (proxy for “patient family physician”) was defined as having at least two consultations with a single GP or at least two consultations with more than one GP working in the same family medicine group*.

e *Usual outpatient psychiatrist was defined as at least having two consultations with a psychiatrist, or, if the patient had only one such consultation, at least two consultations with his/her GP, referred to as collaborative care*.

f *Regarding main medical provider, for the subgroup “both GP and psychiatrist” patients must have had at least one consultation with a psychiatrist and two consultations with his/her GP in outpatient care*.

g *Usual provider continuity index describes the proportion of visits to the usual GP and usual outpatient psychiatrist of the total GP and outpatient psychiatrist visits*.

Table 3 shows comparative results for MD-CPI and MD only on frequency of ED use at 12-month follow-up, controlling for clinical, sociodemographic, and service use variables. Risk of ED use among patients with MD-CPI increased 17% (IRR = 1.17; 95% CI = 1.05–1.30) compared to those with MD only. Substance-related disorders increased the risk by 39% (IRR = 1.39; 95% CI = 1.23–1.58), while severity scores for chronic illnesses ranged from 1 to 3, increasing risk by 39% (IRR = 1.39; 95% CI = 1.17–1.65) for patients with a severity score of 1 and by 40% (IRR = 1.40; 95% CI = 1.17–1.67) for those scoring 3. Patients 65 years+ had 25% (IRR = 0.75; 95% CI = 0.64–0.84) less risk of ED use vs. those 12–24 years old. Emergency department use increased to 25% (IRR = 1.25; 95% CI = 1.12–1.39) among patients having 1–3 consultations with their usual psychiatrist or 1–3 psychosocial interventions in community healthcare centers (IRR = 1.25; 95% CI = 1.14–1.37); while ED use augmented of 31% among patients having 4+ consultations (IRR = 1.31; 95% CI = 1.18–1.46) with their usual psychiatrist and of 27% (IRR = 1.27; 95% CI = 1.14–1.40) among those having 4+ psychosocial interventions in community healthcare centers. High continuity of physician care decreased the risk of t ED use by 23% (IRR = 0.77; 95% CI = 0.69–0.86).

**Table 3:** Negative binomial model comparing groups of patients diagnosed at onset of mental disorders (MD) including substance-related disorders (SRD) with or without chronic physical illnesses (CPI) on frequency of emergency department (ED) visits for mental health reasons at 12-month follow-up to index ED visit in 2014–15.

	**IRR (95% CI)**	Adjusted IRR (95% CI)^*^	***P*-value for adjusted IRR**
**Clinical variables** (2012–13 to index ED visit)			
Patient group with MD and CPI (ref.: patient group with MD only)	1.23 (1.17–1.37)	1.17 (1.05–1.30)	0.004
MD at 2014–15 index ED visit (ref.: no MD)			
Common MD	0.98 (0.91–1.05)	0.98 (0.91–1.05)	0.65
Serious MD	0.99 (0.90–1.08)	0.94 (0.85–1.03)	0.223
SRD vs. no SRD	1.36 (1.20–1.54)	1.39 (1.23–1.58)	<0.001
Elixhauser Comorbidity Index (severity of chronic physical illnesses)^a^			
1 vs. 0	1.49 (1.28–1.74)	1.39 (1.17–1.65)	<0.001
2 vs. 0	1.44 (1.19–1.74)	1.37 (1.11–1.68)	0.003
3+ vs. 0	1.44 (1.23–1.69)	1.40 (1.17–1.67)	<0.001
**Sociodemographic variables** (2014–15)			
Age			
25–44 vs. 12–24 years	0.96 (0.88–1.04)	0.93 (0.85–1.01)	0.111
45–64 vs. 12–24 years	1.03 (0.93–1.13)	0.93 (0.85–1.03)	0.213
65+ vs. 12–24 years	0.87 (0.75–1.01)	0.75 (0.64–0.88)	<0.001
Sex (ref.: women)			
Men	0.99 (0.92–1.06)	1.01 (0.95–1.09)	0.591
**Service use variables** (12-month follow-up to index ED visit in 2014–15)			
Frequency of consultations with usual general practitioner (GP)^b^			
1–3 consultations vs. none	0.98 (0.90–1.07)	1.10 (0.98–1.22)	0.086
4+ consultations vs. none	0.99 (0.91–1.08)	1.10 (0.99–1.24)	0.069
Frequency of consultations with usual outpatient psychiatrist^c^			
1–3 consultations vs. none	1.17 (1.06–1.30)	1.25 (1.12–1.39)	<0.001
4+ consultations vs. none	1.18 (1.07–1.29)	1.31 (1.18–1.46)	<0.001
Frequency of psychosocial interventions in community healthcare centers (excluding GP interventions)			
1–3 consultations vs. none	1.22 (1.12–1.33)	1.25 (1.14–1.37)	<0.001
4+ consultations vs. none	1.34 (1.24–1.46)	1.27 (1.14–1.40)	<0.001
High Usual Provider Continuity Index integrating GP and psychiatrist (continuity of physician care)^d^			
≥0.61 (high continuity of physician care) vs. <0.61 (low continuity of physician care)	0.94 (0.87–1.01)	0.77 (0.69–0.86)	<0.001

*Confidence interval (CI); incidence rate ratio (IRR)*.

* *Adjusted IRR for all variables*.

a *CPI were diagnosed 2 years prior to index ED visit in 2014–15. Elixhauser Comorbidity Index included: chronic pulmonary illnesses, cardiac arrhythmias, tumor w/o metastasis, renal failure, fluid electrolyte illnesses, myocardial infarction, congestive heart failure, metastatic cancer, dementia, cerebrovascular illnesses, neurological illnesses, liver illnesses (excluding alcohol-induced liver disease), pulmonary circulation illnesses, coagulopathy, weight loss, paralysis, AIDS/HIV. Of the 31 illnesses reported in this index, 4 related to MD or SRD categories were excluded. This index was originally developed for mortality, not for ED use or hospitalization, which may have affected findings for this study*.

b *Usual GP (proxy for “patient family physician”) was defined as having at least two consultations with a single GP or at least two consultations with more than one GP working in the same family medicine group*.

c *Usual outpatient psychiatrist was defined as having at least two consultations with a psychiatrist, or, if the patient had only one such consultation, at least two consultations with his/her GP, referred to as collaborative care*.

d *High usual Provider Continuity Index describes the proportion of visits to the usual GP and usual outpatient psychiatrist of total GP and outpatient psychiatrist visits*.

Table 4 presents results for MD-CPI compared with MD only on hospitalization at 12-month follow-up, controlling for the same variables as above. Patients with MD-CPI had 59% (IRR = 1.59; 95% CI = 1.30–1.94) greater risk of hospitalization than those with MD only. Serious MD increased the risk of hospitalization by 62% (IRR = 1.62; 95% CI = 1.36–1.92). Risk of hospitalization for patients with common MD decreased by 37% (IRR = 0.63; 95% CI = 0.53–0.74) and for patients with personality disorders by 32% (IRR = 0.68; 95% CI = 0.51–0.89). Compared with patients 12–24 years old, those 45–64 years were 26% (IRR = 1.26; 95% CI = 1.01–1.56) and those 65+ were 88% (IRR = 1.88; 95% CI = 1.41–2.49) more likely to be hospitalized. Men were at 17% (IRR = 1.17; 95% CI = 1.01–1.36) greater risk for hospitalization than women. Patients who received 1–3 outpatient psychiatric consultations were 1.89 times (IRR = 2.89; 95% CI = 2.29–3.65) more likely to be hospitalized, while the likelihood for those with 4+ consultations increased 3.25 times (IRR = 4.25; 95% CI = 3.47–5.22). By contrast, risk of hospitalization decreased to 36% (IRR = 0.64; 95% CI = 0.52–0.79) for those receiving high continuity of physician care, and to 29% (IRR = 0.71; 95% CI = 0.57–0.88) after 4+ consultations with the usual patient GP.

**Table 4:** Negative binomial model comparing groups of patients at onset of mental disorders (MD) including substance-related disorders (SRD) with or without chronic physical illnesses (CPI) on frequency of hospitalization for mental health reasons at 12-month follow-up to index ED visit in 2014–15.

	**IRR**	Adjusted IRR (95% CI)^*^	***P*-value for adjusted IRR**
**Clinical variables** (2012–13 to index ED visit)			
Patient group with MD and CPI (ref.: patient group with MD only)	2.04 (1.73–2.40)	1.59 (1.30–1.94)	<0.001
MD at 2014–15 index ED visit (ref.: no MD)			
Common MD	0.47 (0.40–0.56)	0.63 (0.53–0.74)	<0.001
Serious MD	3.04 (2.59–3.57)	1.62 (1.36–1.92)	<0.001
Personality disorders	0.64 (0.48–0.85)	0.68 (0.51–0.89)	<0.005
SRD vs. no SRD	1.07 (0.80–1.45)	1.21 (0.92–1.59)	0.169
Elixhauser Comorbidity Index (severity of chronic physical illnesses)^a^			
1 vs. 0	1.40 (0.98–1.99)	1.01 (0.72–1.44)	0.911
2 vs. 0	1.87 (1.26–2.79)	1.06 (0.73–1.45)	0.738
3+ vs. 0	1.97 (1.42–2.73)	1.28 (0.92–1.75)	0.133
**Sociodemographic variables** (2014–15)			
Age			
25–44 vs. 12–24 years	1.13 (0.92–1.40)	1.16 (0.95–1.41)	0.139
45–64 vs. 12–24 years	1.41 (1.13–1.75)	1.26 (1.01–1.56)	0.033
65+ vs. 12–24 years	2.31 (1.76–3.05)	1.88 (1.41–2.49)	<0.001
Sex			
Men vs. women	1.17 (1.00–1.38)	1.17 (1.01–1.36)	0.036
**Service use variables** (12-month follow-up to index ED visit in 2014–15)			
Frequency of consultations with usual general practitioner (GP)^b^			
1–3 consultations vs. none	0.84 (0.69–1.03)	0.84 (0.68–1.02)	0.100
4+ consultations vs. none	0.65 (0.53–0.80)	0.71 (0.57–0.88)	0.002
Frequency of consultations with usual outpatient psychiatrist^c^			
1–3 consultations vs. none	2.15 (1.74–2.65)	2.89 (2.29–3.65)	<0.001
4+ consultations vs. none	4.03 (3.40–4.78)	4.25 (3.47–5.22)	<0.001
High Usual Provider Continuity Index integrating GP and psychiatrist (continuity of physician care)^d^			
≥0.61 (high continuity of physician care) vs. <0.61 (low continuity of physician care)	1.13 (0.96–1.33)	0.64 (0.52–0.79)	<0.001

*Confidence interval (CI); incidence rate ratio (IRR)*.

* *Adjusted IRR for all variables*.

a *CPI were diagnosed 2 years prior to index ED visit in 2014–15. Elixhauser Comorbidity Index included: chronic pulmonary illnesses, cardiac arrhythmias, tumor w/o metastasis, renal failure, fluid electrolyte illnesses, myocardial infarction, congestive heart failure, metastatic cancer, dementia, cerebrovascular illnesses, neurological illnesses, liver illnesses (excluding alcohol-induced liver disease), pulmonary circulation illnesses, coagulopathy, weight loss, paralysis, HIV/AIDS. Of the 31 illnesses reported in this index, 4 related to MD or SRD were excluded. This index was originally developed for mortality, not for ED use or hospitalization, which may have affected findings for this study*.

b *Usual GP (proxy for “patient family physician”) was defined as having at least two consultations with a single GP or at least two consultations with more than one GP working in the same family medicine group*.

c *Usual outpatient psychiatrist was defined as having at least two consultations with a psychiatrist, or, if the patient had only one such consultation, at least two consultations with his/her GP, referred to as collaborative care*.

d *High usual Provider Continuity Index describes the proportion of visits to the usual GP and usual outpatient psychiatrist of total GP and outpatient psychiatrist visits*.

## Discussion

This study was original in measuring frequencies of both ED use and hospitalization for MH reasons, comparing patients with MD-CPI and those with MD only, and controlling for multiple clinical, sociodemographic, and service use variables. Overall, we found that one patient in four had MD-CPI. As patients recruited from ED tend to be affected with serious or complex MD, and most in this sample also experienced material or social deprivation, high prevalence of MD-CPI was expected. As well, most patients had used ED at least once by 12-month follow-up, if not several times, while nearly one fourth were hospitalized.

Patients with MD-CPI were more likely to make frequent use of ED and to be hospitalized for MH reasons than those with MD only during the 12-month follow-up period, confirming hypothesis one. Previous studies found that frequent psychiatric ED users were often affected by co-occurring CPI (7, 14). A recent systematic review also identified higher risk of psychiatric hospitalization among patients with MD-CPI (17). The present study was however one of the first to estimate levels of risk for ED use and hospitalization for MH reasons among patients with MD-CPI, compared to patients with MD only. Overall, the increased risk for ED use was significant, but modest (15%), while hospitalization risk was substantially elevated at 63%.

The findings also partially confirm the second hypothesis that having more serious medical conditions, including SRD or severe CPI, would increase ED use, and that serious MD would increase hospitalization rates. Patients with SRD are known to be high ED users (55, 56) and to use few outpatient services in addition to low treatment compliance or motivation (57, 58). Severity of CPI was found to increase the risks of ED use and hospitalization for medical reasons (17, 24, 33). Studies have also identified CPI as key determinants of high ED use for MH reasons, and serious MD an added impetus for hospitalization (59, 60). Other studies reported that patients with MD-CPI were less likely than others to receive care for CPI from their GP (61, 62), suggesting that they may have used psychiatric ED for medical issues. Studies on determinants of hospitalization for medical reasons found a relationship between personality disorders and low hospitalization (59, 63). This may be partially explained by negative attitudes among hospital staff and stigmatization experienced by patients with personality disorders, and particularly borderline personality disorder, who are viewed as difficult patients, often manipulative or violent (64, 65). Hospitalization is usually considered inappropriate for these patients, except in cases of acute crisis requiring short-stay admission (64). Treatments other than hospitalization are recommended for patients with personality disorders, including outpatient psychiatric treatment (66) or psychosocial treatments (67, 68). The finding that risk of hospitalization decreased among patients with common MD is explained by research showing the effectiveness of primary care treatment for adjustment, anxiety and depressive disorders (69, 70), and that these conditions require less frequent hospitalization than serious MD.

Regarding the third hypothesis related to quality of services, only high continuity of physician care protected against both ED use and hospitalization, while intensive care from the usual GP prevented hospitalization. High continuity of physician care is a key recovery indicator, particularly among patients with MD, CPI, or co-occurring diagnoses (71, 72). Previous studies have demonstrated that higher continuity of care is associated with less frequent ED use (8, 73) and hospitalization (74). Receiving intensive care from GP (4+ consultations yearly) after onset of MD, another continuity of care measure and key indicator for quality of care and patient recovery (75, 76), was found to decreased frequency of hospitalization. It is possible that access to GP for consultations by patients was not sufficiently rapid that their use of ED for MH problems could be avoided or reduced. Patients with MD or both MD-CPI often face crisis situations involving psychological distress or suicidal behaviors, which may also exacerbate co-occurring problems. As such, close follow-up by GP may have helped patients avoid decompensation, while supporting their adherence to treatment and motivation to seek care, as well as protecting them against hospitalization. Some 50% of patients in the sample also reported making no GP consultations and 4 of 10 patients had no medical provider, pointing to important unresolved issues involving access to physician care for this population.

Surprisingly, the study revealed that receiving more intensive outpatient care from psychiatrists increased the risks of both frequent ED use and hospitalization. This result may point to a certain lack of appropriateness or diversity in outpatient psychiatric treatments commensurate with the needs of patients affected by very complex and serious conditions requiring highly specialized care. Previous research has shown similar results (14, 59). Community healthcare centers also offered psychosocial interventions to more vulnerable individuals who which perhaps had led to these patient higher risks of frequent ED use. These organizations also provided more treatments for individuals with common MH problems, which may explain the lack of association with risk of hospitalization.

Among sociodemographic variables, age was associated with both ED use and hospitalization, while sex was associated with hospitalization only. Compared with patients in the 12–24 age group, those 65+ were less likely to use ED for MH reasons, possibly because MD are often underdiagnosed or undertreated among older patients, whereas CPI are more commonly addressed (77, 78). Yet patients 65+, as well as those in the 45–64 age group, were at relatively greater risk for hospitalization. Sources of care other than hospitalization are usually preferred for younger patients, due to perceived stigma (79, 80). Moreover, the finding that men had an elevated risk of hospitalization may be explained by research suggesting that men generally use services as a last resort, once their MH problems become quite serious (81). Men also use primary care less than women (82).

This study had several limitations. First, Quebec medical administrative databases were developed primarily for financial purposes, and as such the results represent only a proxy for patient needs. Second, key data including ethnicity, medication compliance, side-effects of anti-psychotic medications, and use of psychosocial hospital teams, community MH organizations or psychologists in private practice that may have potentially impacted frequency of ED use and hospitalization were not available from these study databases. Third, use of the ICD-9 codification and not ICD-10 from the RAMQ database, and use of the Elixhauser Comorbidity Index originally developed for mortality rather than ED use and hospitalization may have had underestimated the impact of CPI on study results (44, 46). Finally, findings of the study may not be generalizable to patients with MD who do not use ED, or to patients living in rural areas or in countries without a public healthcare insurance system.

## Conclusions

Overall, patients with MD-CPI were more likely than those with MD only to experience frequent ED use and hospitalization for MH reasons. Findings also confirmed that patients with more severe medical conditions who received intensive specialized MH care were at greater risk for frequent ED use and hospitalization. Older patients were at less risk for ED use, but these patients, as well as men, were more frequently hospitalized. Higher continuity of physician care protected against frequent ED use and hospitalization. Strategies such as assertive community treatment, intensive case management, integrated co-occurring treatment, home treatment, and shared care between psychiatrists and primary care services may be implemented to improve the adequacy of care for patients with MD-CPI or for those with MD only whose clinical profiles are relatively more severe. Prevention and outreach strategies should also be strongly promoted to reduce frequent ED use and hospitalization among older age groups, and particularly among men.

## Data Availability Statement

The raw data supporting the conclusions of this article will be made available by the authors, without undue reservation.

## Ethics Statement

The studies involving human participants were reviewed and approved by Douglas Mental Health Institute. Written informed consent for participation was not required for this study in accordance with the national legislation and the institutional requirements.

## Author Contributions

LG, M-JF, and XM designed the analytic plan for the article. LG produced the quantitative analyses and tables. LG, GG, and M-JF wrote the article. XM reviewed the manuscript prior to submission. All authors thus contributed to the article and approved the submitted version.

## Funding

This study was funded by the Canadian Institutes of Health Research (CIHR, Grant Number 8400997).

## Conflict of Interest

The authors declare that the research was conducted in the absence of any commercial or financial relationships that could be construed as a potential conflict of interest.

## Publisher's Note

All claims expressed in this article are solely those of the authors and do not necessarily represent those of their affiliated organizations, or those of the publisher, the editors and the reviewers. Any product that may be evaluated in this article, or claim that may be made by its manufacturer, is not guaranteed or endorsed by the publisher.

## Abbreviations

AIC: Akaike's Information Criterion
BIC: Bayesian Information Criterion
BDCU: emergency department database *(Banque de Données Commune des Urgences*)
ED: emergency departments
GP: general practitioners
ICC: Intraclass Correlation Coefficient
ICD: International Classification of Diseases
MED-ECHO: hospitalization database (*Maintenance et Exploitation des Données pour l'Étude de la Clientèle Hospitalière*)
MD: mental disorders
SRD: substance-related disorders
RAMQ: Quebec health insurance regime (*Régie de l'Assurance Maladie du Québec*).
